# Naringenin Impairs Two-Pore Channel 2 Activity And Inhibits VEGF-Induced Angiogenesis

**DOI:** 10.1038/s41598-017-04974-1

**Published:** 2017-07-11

**Authors:** Irene Pafumi, Margherita Festa, Francesca Papacci, Laura Lagostena, Cristina Giunta, Vijay Gutla, Laura Cornara, Annarita Favia, Fioretta Palombi, Franco Gambale, Antonio Filippini, Armando Carpaneto

**Affiliations:** 1 Department of Anatomy, Histology, Forensic Medicine and Orthopaedics, Unit of Histology and Medical Embryology, Sapienza University of Rome, 16 Via A. Scarpa, 00161 Rome, Italy; 2 Department of Biosciences, University of Milano, Via Celoria, 26, 20133 Milan, Italy; 3 Institute of Biophysics - CNR, Via De Marini 6, 16149 Genoa, Italy; 4 DISTAV (Department of Earth, Environment and Life Sciences) of the University of Genoa, Corso Europa 26, 16132 Genoa, Italy; 5 Nucleic Acids Laboratory, Institute of Molecular Biology and Pathology, National Research Council (IBPM-CNR) and Dept. of Biology and Biotechnologies, Sapienza University of Rome, Rome, Italy

## Abstract

Our research introduces the natural flavonoid naringenin as a novel inhibitor of an emerging class of intracellular channels, Two-Pore Channel 2 (TPC2), as shown by electrophysiological evidence in a heterologous system, i.e. Arabidopsis vacuoles lacking endogenous TPCs. In view of the control exerted by TPC2 on intracellular calcium signaling, we demonstrated that naringenin dampens intracellular calcium responses of human endothelial cells stimulated with VEGF, histamine or NAADP-AM, but not with ATP or Angiopoietin-1 (negative controls). The ability of naringenin to impair TPC2-dependent biological activities was further explored in an established *in vivo* model, in which VEGF-containing matrigel plugs implanted in mice failed to be vascularized in the presence of naringenin. Overall, the present data suggest that naringenin inhibition of TPC2 activity and the observed inhibition of angiogenic response to VEGF are linked by impaired intracellular calcium signaling. TPC2 inhibition is emerging as a key therapeutic step in a range of important pathological conditions including the progression and metastatic potential of melanoma, Parkinson’s disease, and Ebola virus infection. The identification of naringenin as an inhibitor of TPC2-mediated signaling provides a novel and potentially relevant tool for the advancement of this field of research.

## Introduction

Naringenin (Nar) (5,7-dihydroxy-2-(4-hydroxyphenyl)chroman-4-one) is one of the main flavonoids present in the human diet. Epidemiological studies have demonstrated that the consumption of vegetables and fruit with a high Nar content, such as citruses and tomatoes or their food products, is associated with a reduced incidence of metabolic and chronic-degenerative diseases^1, 2^. Following this kind of evidence, several *in vitro* studies have shown that Nar interacts with various cellular pathways, highlighting its potential as an antioxidant, antinflammatory, chemopreventive and antidegenerative agent^3^; moreover, Nar was shown to exert anti-angiogenic effects in the avian chorio-allantoid membrane model^4^ while this manuscript was being prepared. Naringenin lowers the plasma and hepatic cholesterol concentrations by suppressing HMG-CoA reductase and ACAT in rats fed a high-cholesterol diet^5^. Pre-clinical studies have revealed the potential of Nar and of its precursor naringin in the treatment of metabolic and cardiovascular disorders, including hyperlipidemia, hypertension, cardiac toxicity, hyperglycemia and diabetes, hepatic steatosis and atherosclerosis^2^; its therapeutic use to combat various kinds of cancer has also been envisaged^3^. Indeed, Nar exerts chemopreventive and anticancer activity by blocking the progression and the formation of metastasis in various experimental models of oral^6^, melanoma^7^, breast^8, 9^, colon^10^, lung^11^ and liver^12^ cancer. It acts by upregulating many different cell survival proteins or arresting the cell cycle, by inducing p53-dependent apoptosis or inhibiting inflammatory processes and, in some cases, by exploiting all of these mechanisms^13–15^. Despite the wealth of evidence on Nar efficacy, a comprehensive view of its mechanism of action is still lacking, as is the experimental evidence needed to bridge the gap between multiple cellular targets and biological response. It is worth noting that Nar is known to affect the activity of different calcium-activated or calcium-permeable ion channels. At a concentration of 100 μM, Nar induces a threefold increase in the currents mediated by large conductance Ca^2+^-activated K^+^ (BKCa) channels, which can be closely correlated with the significant vasorelaxant effect mediated by Nar on rat endothelium-denuded vessels^16^. Similar conclusions have been drawn by other authors: the relaxant effect of Nar on rat colon smooth muscle has been attributed to the direct activation of BKCa channels, resulting in hyperpolarization of smooth muscle cells, which in turn reduces the Ca^2+^ influx through voltage-dependent calcium channels^17^. Nar is a powerful inhibitor (IC50 of 0.5 μM) of the melastatin-related transient receptor potential TRPM3 belonging to the TRP family, a calcium-permeable non-selective cation channel expressed in various neural and non**-**neural tissues, activated by neurosteroids and heat^18^. Since TRPM3-deficient mice display an impaired perception of noxious heat, the inhibition of TRPM3 may represent a novel tool for analgesic therapy^19^. Another member of the TRP family, TRPP2 or polycystin-2, a Ca^2+^-permeable non-selective cation channel located in the endoplasmic reticulum and in the primary cilium, is modulated by Nar concentrations ranging from 50 to 200 μM^20^. This channel is implicated in the development of autosomal dominant polycystic kidney disease (ADPKD). It has been suggested that Nar may activate TRPP2 to cause Ca^2+^ influx and a decrease in cellular proliferation, thereby providing a novel therapeutic approach to ADPKD^20^. It is noteworthy that all the aforementioned effects are fully reversible upon Nar removal from the extracellular solution. Prompted by evidence pointing to Ca^2+^ as a possible mediator of biological responses to Nar, we explored the involvement of an emerging family of intracellular channels known as two-pore channels (TPCs). In humans, two TPC members are present, namely hTPC2, which targets lysosomes, and hTPC1, which is more widely distributed within the endolysosomal system despite being expressed predominantly in endosomes^21, 22^. The only member of the TPC family present in the model plant *Arabidopsis thaliana* is AtTPC1, which has been shown^23^ to encode the so-called, previously described, SV current^24^. The structure of AtTPC1 was very recently revealed by X-ray crystallography^25–27^. The structure of TPCs is dimeric with each subunit being formed by two covalently-linked, shaker-like domains^28^. From the functional point of view, hTPC2 is gated by the powerful Ca^2+^ mobilizer Nicotinic Acid Adenine Dinucleotide Phosphate (NAADP)^21, 29, 30^, although the binding of NAADP is likely to be mediated by a small accessory protein^31–33^ that has yet to be identified. Besides NAADP, hTPC2 is also directly activated by the phosphoinositide PI(3,5)P_2_^34–36^. TPC2 knock out mice, when fed with a Western-type diet rich in cholesterol, suffered of non-alcoholic fatty liver hepatitis with enhanced hepatic cholesterol accumulation and hyperlipoproteinaemia^37^; however, TPC2 deficient mice fed with a standard diet were identical to wild type^37^. Interestingly, hTPC2 has recently been related to a number of pathologies^38^, such as neurodegenerative Parkinson disease^39^ and viral infection (i.e. Ebola virus)^40^, and has emerged as an important player in neoangiogenesis^41^, which is highly relevant to the purposes of this work*. In vitro* experiments in HUVECs, using either a selective NAADP antagonist (Ned-19) or anti-TPC2 shRNA, revealed a drastic inhibition of angiogenic responses to VEGF following the significant reduction in intracellular Ca^2+^ mobilization. Moreover, *in vivo* experiments showed that Ned-19 inhibits VEGF-induced vessel formation in matrigel plugs, which also failed to occur in TPC2 −/− mice but was unaffected in TPC1 −/− mice^41^.

The present study investigates the ability of Nar to directly bind TPC2 and inhibit its activation as well as to specifically abolish the NAADP/TPC2-dependent mobilization of Ca^2+^ induced by VEGF. Interestingly, this inhibition is paralleled by an impairment in angiogenic responses to VEGF both *in vitro* and in an established *in vivo* murine model. Taken as a whole, these data identify TPC2 as a novel and important target for Nar, shedding new light on its mechanism of action.

## Results

### Naringenin reversibly impairs the functional activity of the human TPC2 channel

**Figure 1:**
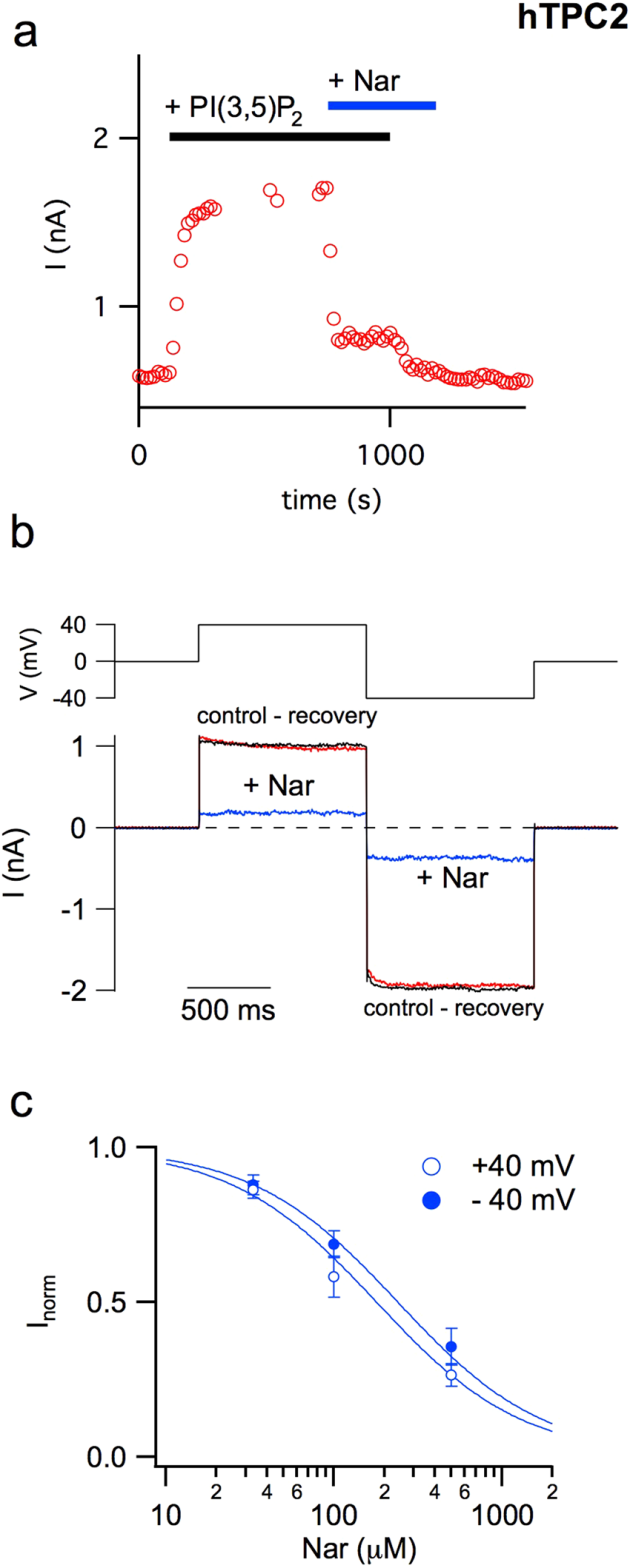
Naringenin inhibits the activity of the human TPC2 channel. (**a**) Time course of current amplitude recorded in response to bath application of 330 nM PI(3,5)P_2_ (upper black bar) before and after adding 500 μM Nar (upper blue bar). Each point represents the steady-state current at +40 mV. (**b**) Currents mediated by hTPC2 and induced by the application of 330 nM PI(3,5)P_2_ in the cytosolic bath solution were recorded at +40 and −40 mV (shown in the upper voltage profile) in the absence or presence of 500 μM cytosolic Nar. For the sake of clarity, background currents in the absence of the phosphoinositide were directly subtracted. (**c**) Dose–response analysis of Nar inhibition. I_norm_, the normalised current, was obtained by the ratio of currents recorded respectively in the presence and absence of cytosolic Nar. Both at +40 and −40 mV, normalised currents at different Nar concentrations were fitted with a Michaelis–Menten function (continuous lines). Data from 3 (Nar 33 μM), 6 (Nar 100 μM) and 9 (Nar 500 μM) different vacuoles were shown as mean ± s.e.m. Values at positive and negative voltages, at a defined Nar concentration, were not significant (P > 0.2); values at different Nar concentrations and at a defined voltage (+40 or −40 mV), were statistically significant (at least P < 0.01).

Flavonoids have the ability to permeate biological membranes by passive diffusion^42, 43^ and, as a consequence, to reach subcellular compartments, such as lysosomes where hTPC2 is localized. This prompted us to investigate whether Nar, a main flavonoid present in human diet, was effective in modulating the activity of this channel. To test this hypothesis, we used a novel heterologous system recently developed for the expression and functional characterization of animal intracellular channels and transporters^35, 44, 45^. We transiently transformed protoplasts isolated from a mutant of *Arabidopsis thaliana* lacking the endogenous TPC with hTPC2 equipped with an EGFP fused to its C-terminus^35^. We applied the patch-clamp technique to vacuoles in which GFP fluorescence was clearly detectable on the tonoplast. The addition of 330 nM PI(3,5)P_2_ in the external (cytosolic) solution induced robust hTPC2-mediated currents, as shown in Fig. 1a where stationary currents recorded at +40 mV were plotted versus time. When 500 μM Nar was also added in the cytosolic solution a strong decrease in the activity of hTPC2 currents was evident. Figure 1b shows control and recovery currents (ΔI) recorded in the presence of PI(3,5)P_2_ subtracted by the correspondent background recorded without the phosphoinositide. Upon Nar application, currents were drastically reduced at both +40 and −40 mV voltages. Even if recorded in symmetrical concentration of sodium, due to a voltage-dependent block by cytosolic magnesium^36^, the currents at +40 mV were smaller than those recorded at −40 mV. Interestingly, Nar inhibition was fully reversible (Fig. 1b). The dose-response was well described by a Michaelis-Menten hyperbolic function (Fig. 1c), with apparent affinity constants of 180 ± 20 μM at +40 mV and of 240 ± 40 μM at −40 mV, therefore not significantly different.

### The activity of human TPC1 channel is decreased by cytosolic naringenin

**Figure 2:**
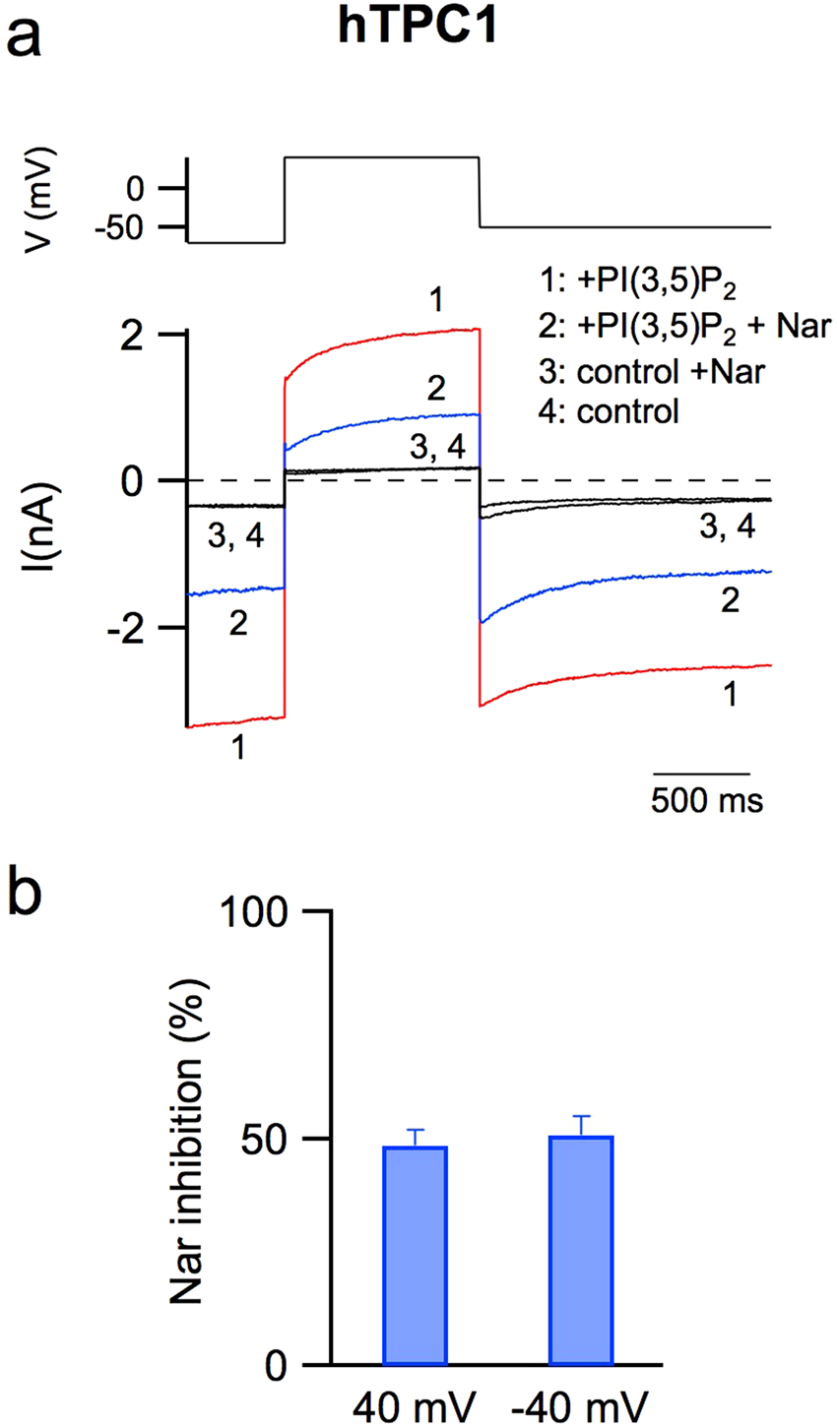
The activity of the human TPC1 channel is inhibited by naringenin. (**a**) Currents (lower panel) recorded in control conditions (trace 4) or adding in the cytosolic bath solution: 90 nM PI(3,5)P_2_ (trace 1), 90 nM PI(3,5)P_2_ and 500 μM Nar (trace 2), 500 μM Nar (trace 3). In the upper panel the voltage profile is shown. (**b**) Percentage of current inhibition induced by 500 μM Nar added in the cytosolic solution at −40 and + 40 mV. Data from 6 different vacuoles, shown as mean ± s.e.m., were not statistically significant (P > 0.6).

We wondered if Nar could modulate the other member of the TPC family in humans, namely hTPC1. We therefore performed a similar approach as for hTPC2 and expressed hTPC1 fused to an EGFP at its C-terminus in *Arabidopsis Thaliana* vacuoles lacking the endogenous TPC1. Suppl. Fig. 1a, left panel, shows that the background currents recorded in the absence of PI(3,5)P_2_ are very small in comparison to those elicited by the addition of the phosphoinositide (right panel). We chose a concentration of PI(3,5)P_2_ equal to 90 nM and therefore near to the saturation value since the apparent binding constant in this experimental condition was about 20 nM^46^; in comparison the affinity binding constant of PI(3,5)P_2_ for hTPC2 is about 140 nM^35^. The current-voltage relationship in the absence (empty symbols) and in the presence (filled symbols) of PI(3,5)P_2_ was plotted in Suppl. Fig. 1b. In a recent paper^46^, we demonstrated that the functionality of hTPC1 was strongly affected by both cytosolic and luminal Ca^2+^, namely an increase in cytosolic and luminal Ca^2+^ induced an increase and a decrease of the activity of the channel, respectively. Here, our standard pipette and ionic bath solutions (see Materials and Methods) allowed us to record hTPC1-mediated currents at both negative and positive potentials and therefore to evaluate the effect of Nar at different voltages. In Suppl. Fig. 1b, at positive voltages, a voltage dependent inhibition of the hTPC1-induced currents due to the presence of cytosolic magnesium was also apparent^46^; a similar effect was recorded for hTPC2 in Fig. 1b and by other authors^36^. Figure 2a showed that the addition to the cytosolic solution of 500 μM Nar (trace 2) induced a significant decrease of hTPC1-elicited currents (trace 1) at both negative and positive voltages and had almost no effect in control conditions (compare trace 3 and 4). The inhibition of hTPC1 by Nar was quantified in the histogram of Fig. 2b; the effect was not voltage-dependent. Moreover, we verified that Nar decreased the activity of the endogenous *Arabidopsis* TPC1 (Suppl. Fig. 2) showing that the inhibition of TPC channels by Nar was a mechanism conserved during evolution.

### Naringenin inhibits VEGF-dependent calcium mobilization in a dose-dependent manner

**Figure 3:**
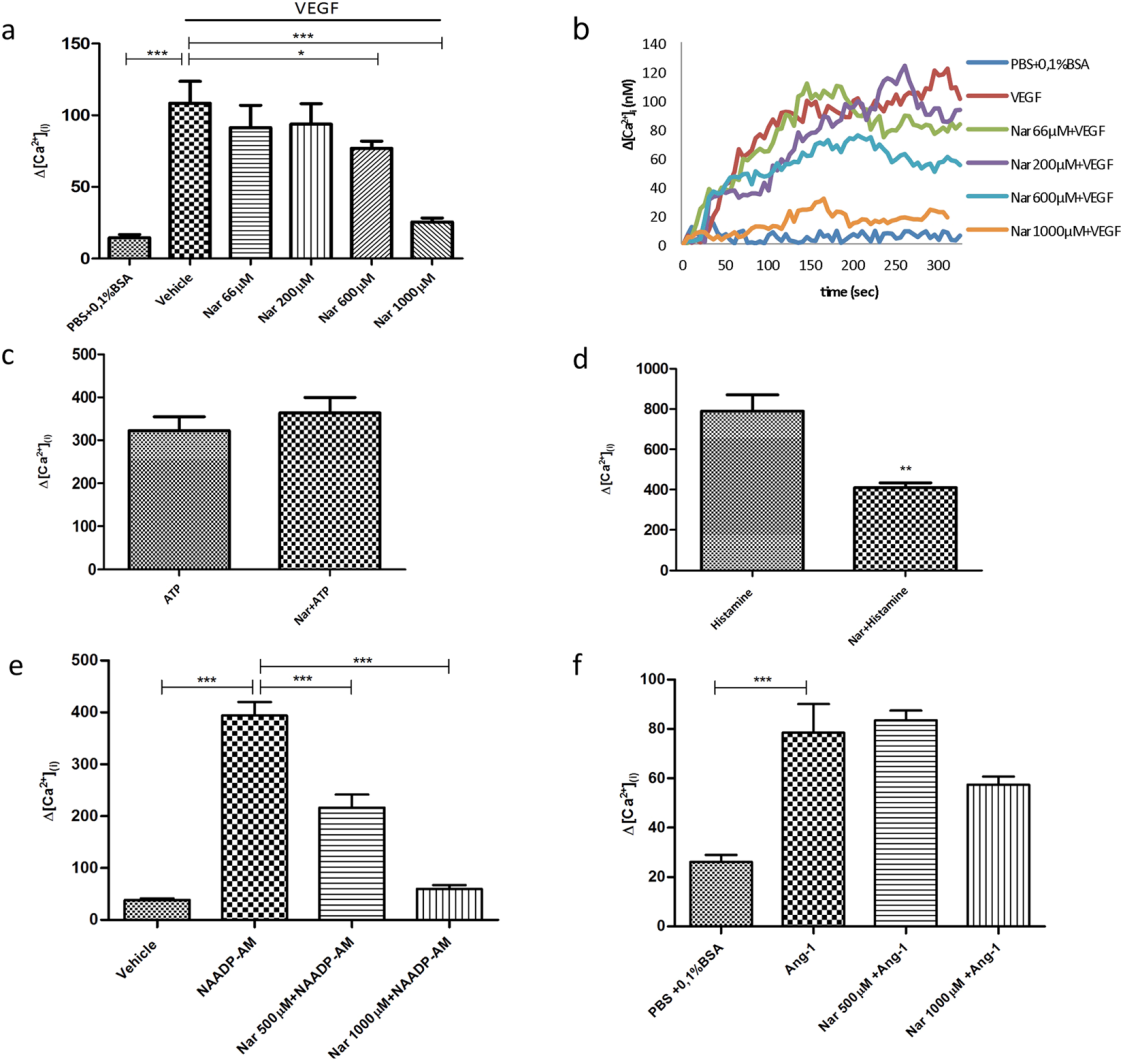
Naringenin inhibits Ca^2+^ release from HUVEC acidic stores under VEGF or NAADP-AM stimulation. Ca^2+^ imaging experiments. (**a**,**b**) Cells were pretreated for 30 min with different concentrations of Nar and then stimulated with 100 ng/ml VEGF; (**a**) Bar chart showing maximum Ca^2+^ concentrations; (**b**) Changes in Ca^2+^ levels shown as representative traces. (**c**,**d**) Cells were pretreated with 1000 µM Nar for 30 min, then stimulated with (**c**) 10 µM ATP (negative control) or (**d**) 100 µM histamine (positive control). (**e**,**f**) Cells were pretreated with 500 µM or 1000 µM Nar and then stimulated with (**e**) 500 nM NAADP-AM or (**f**) 100 ng/ml Ang-1. Data in bar charts were from 3 independent experiments, *n* = 41–180 cells. *P < 0.05; **P < 0.01; ***P < 0.001. Neither 500 µM Nar nor 1000 µM Nar significantly inhibited Ang-1 mediated Ca^2+^ release (P > 0.2).

We have previously demonstrated that in HUVECs stimulated with VEGF intracellular Ca^2+^ mobilization is impaired when cells are pretreated with the NAADP antagonist Ned-19^41^. To characterize the possible inhibitory effect of Nar on the regulation of VEGF-dependent Ca^2+^ signaling, Ca^2+^ imaging experiments were performed on cells pretreated for 30 min with different concentration of Nar and then stimulated with 100 ng/ml VEGF. Ca^2+^ mobilization was significantly reduced in a dose dependent manner (Fig. 3a,b) without substantial impairment of VEGFR2 phosphorylation (Suppl. Fig. 3), hence downstream of receptor activation. Choosing 1000 µM Nar for its powerful inhibitory effect we tested the specificity of this response for NAADP/TPC2-dependent Ca^2+^ signaling using positive and negative control agonists. Nar treatment was observed to block histamine-evoked Ca^2+^ release known to be NAADP/TPC2-dependent^47^ (Fig. 3d), but failed to block Ca^2+^ response to ATP (Fig. 3c), known to be IP_3_-dependent and NAADP–independent. In addition, to more directly assess the specific inhibitory effect of Nar treatment on TPC2, HUVECs were stimulated with the cell permeant form of NAADP, the selective TPC2 agonist. Ca^2+^ imaging experiments with 500 nM NAADP-AM were performed in a Ca^2+^-free buffer so as to monitor only internal mobilization. The histogram in Fig. 3e shows that Nar treatment significantly impairs intracellular Ca^2+^ release at both 1000 µM and 500 µM. Nar selectivity on NAADP/TPC2 pathway was further tested by the use of Ang-1, an angiogenic agonist known to elicit NAADP-independent Ca^2+^ mobilization^48^. Figure 3f and Suppl. Fig. 4a show that Ca^2+^ mobilization stimulated by Ang-1 is not inhibited respectively by Nar and by Ned-19. Collectively, these data are a strong indication that the inhibition of hTPC2 by Nar is responsible for the reduced Ca^2+^ release.

### The role of naringenin in the angiogenic process *in vitro* and *in vivo*

**Figure 4:**
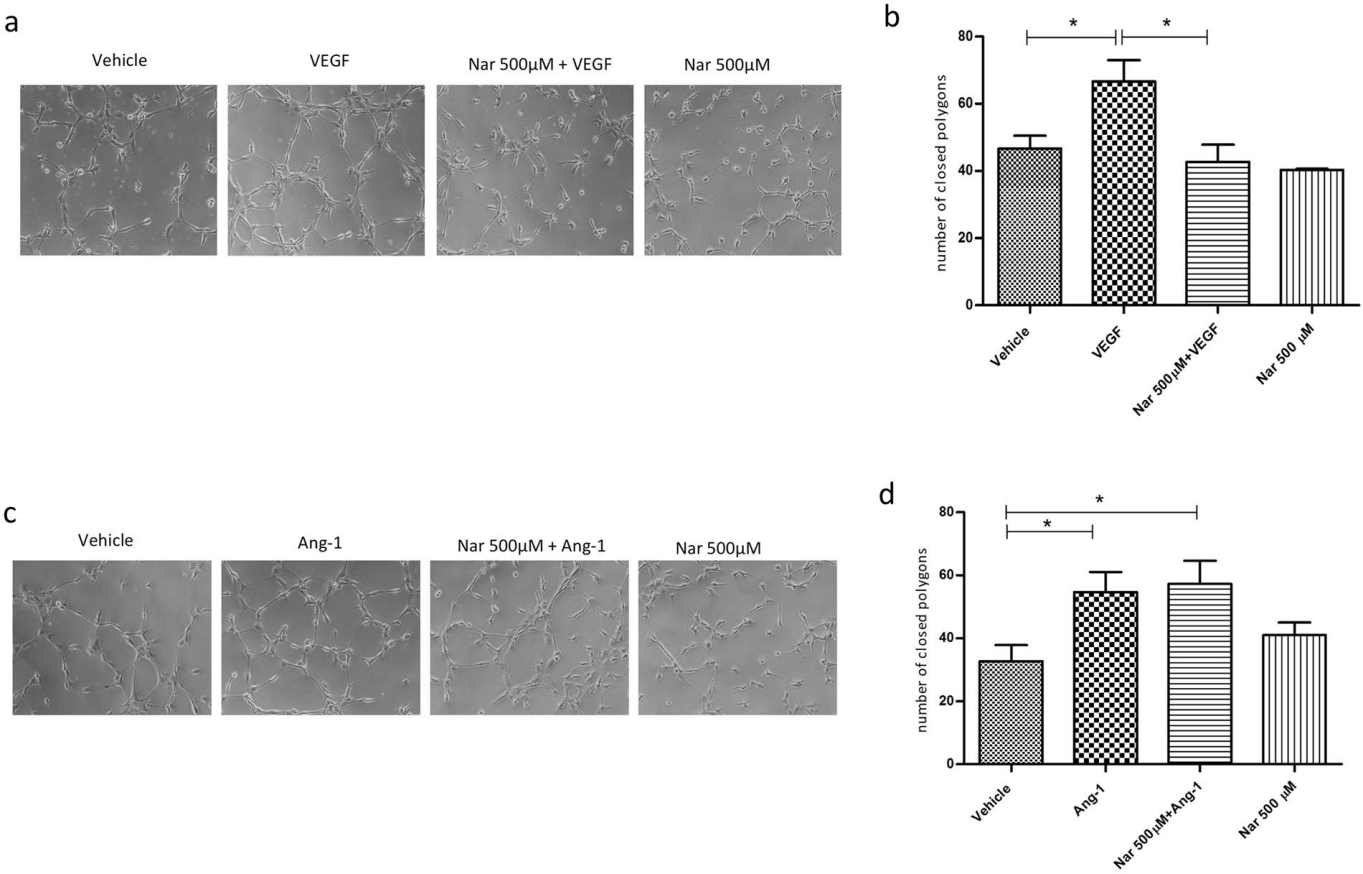
Naringenin impairs VEGF-induced vessel formation *in vitro*. (**a**) Representative images of one of three independent experiments. HUVECs were plated in Matrigel-coated dishes and incubated for 3–4 h in EBM-2 + 2% FBS supplemented or not with VEGF or Nar, or in medium containing both VEGF and Nar. (**c**) Representative images of one of three independent experiments. HUVECs were plated in Matrigel-coated dishes and incubated for 2–3 h in EGM-2 supplemented or not with Ang-1 or Nar, or in medium containing both Ang-1 and Nar. (**b**,**d**) Quantitative evaluation of tube formation as the number of closed polygons formed in 6 fields for each experimental condition for VEGF (**b**) and Ang-1 (**d**). Data in bar charts represent mean ± s.e.m. of three independent experiments. *P < 0.05.

The formation of capillary-like tubes *in vitro* is commonly considered as representative of later, differentiative, steps of angiogenesis and is tested to assess the efficiency of compounds as pro- or antiangiogenic. HUVECs plated onto matrigel matrix adhere, migrate and, within a few hours, differentiate into capillary-like structures, a process enhanced by VEGF. An approximate estimate of the efficiency of this process can be evaluated by the extent of cellular network formation, whereby cells first align to form linear segments, which subsequently interconnect to form closed polygonal structures. As shown in Fig. 4a,b, the number of closed polygons formed in cells stimulated with VEGF in the presence of 500 µM Nar was significantly reduced (*P < 0.01) compared with samples stimulated with VEGF alone. The inhibitory effect of Nar on the *in vitro* angiogenic process was seen also with 1000 µM Nar (Suppl. Fig. 5). Interestingly, the ability of HUVECs to form closed polygons under Ang-1 stimulation was neither affected by treatment with 500 µM Nar (Fig. 4c,d) nor with 100 µM Ned-19 (Suppl. Fig. 4b). These findings indicate that in the regulation of capillary-like formation *in vitro* Nar specifically inhibits NAADP/TPC2-mediated Ca^2+^ signalling.

**Figure 5:**
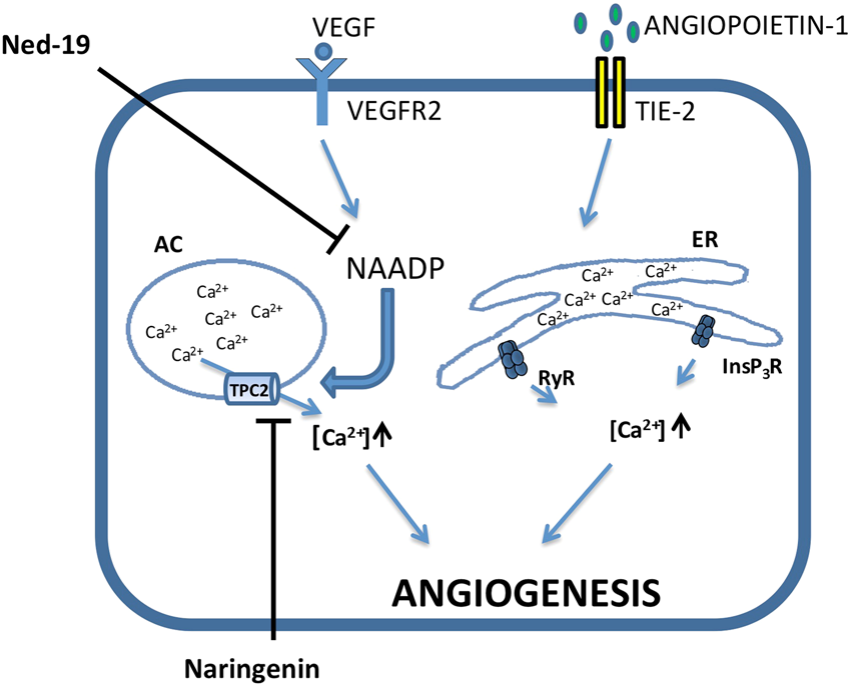
Simplified representation of differential Ca^2+^ signalling pathways involved in the control of angiogenesis by VEGF and Ang-1. Calcium signalling is inhibited by Nar when mediated by TPC2 but not by RyR/InsP_3_R. AC: acidic compartments, ER: endoplasmic reticulum.

The specific role of Nar in the control of VEGF-induced angiogenesis is schematically represented in Fig. 5, based on present and previous data.

**Figure 6:**
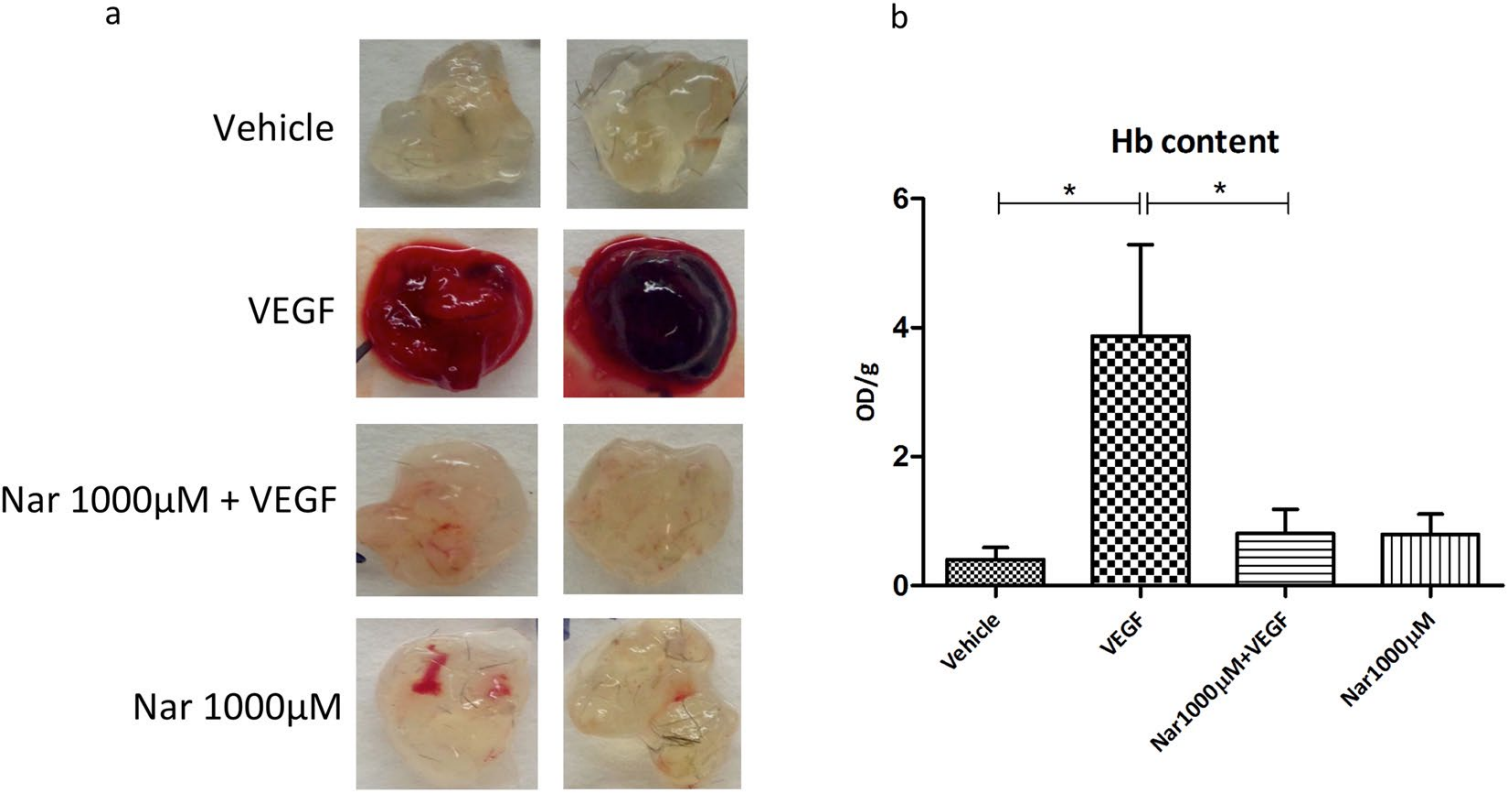
Naringenin impairs *in vivo* vascularization induced by VEGF in C57BL/6 mice. *In vivo* vessel formation was assessed after subcutaneous injection of 5 weeks old male/female C57BL/6 mice with Matrigel plugs containing either vehicle or VEGF or VEGF plus 1000 μM Nar. Five days after injection the mice were sacrificed and plug vascularization was evaluated both macroscopically, as shown in two representative images (**a**) and as hemoglobin content expressed as absorbance (OD)/1 g matrigel plug (**b**); values from three independent experiments are expressed as mean ± s.e.m. *P < 0.05. *n* = 14–15 plugs for each experimental condition.

The involvement of NAADP-mediated Ca^2+^ signaling in the regulation of the *in vivo* angiogenic process has been previously demonstrated in a murine model^41^. In the present study, to assess the inhibitory effect of Nar on *in vivo* angiogenesis, matrigel plug assays were performed. Five days after subcutaneous injections of plugs in C57BL/6 mice, the extent of plug vascularization under different experimental conditions was evaluated by measuring the hemoglobin (Hb) content. As macroscopically apparent (Fig. 6a), the plugs containing VEGF, but not those containing VEGF plus Nar or vehicle alone, underwent intense vascularization. Hb content in the plugs containing VEGF plus Nar was significantly lower than in the plugs containing VEGF alone (Fig. 6b). Conversely, *in vivo* vascularization induced by Ang-1 was not significantly affected by Nar (Suppl. Fig. 6).

## Discussion

In this work we show that intracellular TPC channels are a target for Nar and that this flavonoid can specifically inhibit the ability of TPC2 to elicit Ca^2+^ release from acidic compartments in NAADP-mediated responses to different agonists i.e. VEGF and histamine. We have previously demonstrated in endothelial cells that TPC2 plays an essential role in the intracellular Ca^2+^ increase elicited by histamine and VEGF^41, 47, 49^, which in turn affects Von Willebrand factor secretion and pathophysiological neoangiogenesis respectively.

TPCs are members of the voltage-gated ion channel superfamily and localize in acidic Ca^2+^ stores; in particular TPC2, which has a more restricted subcellular localization, is expressed predominantly in late endosomes and lysosomes^21^. The fact that TPCs have been implicated in different pathophysiological processes^38, 50^ has led to numerous studies being conducted on their mechanism of action in an attempt to identify a drug that regulates TPC activity. In this study, we used electrophysiological tests to investigate the effects of the flavonoid Nar on intracellular TPC channels and assessed whether VEGF action, which relies upon TPC2 as a hub for intracellular signaling, was consequently affected. We recently developed a heterologous system for functional expression of animal TPC channels, namely plant vacuoles from Arabidopsis knock-out plants lacking endogenous TPC channels^35^. When we expressed hTPC2 in this TPC-free model lysosome, we found that hTPC2 share a similar set of inhibitors^35^, such as the generic ion channel blocker zinc, the Ca^2+^channel inhibitor verapamil and the antibiotic neomycin, with the plant TPC^51^. Accordingly, here we discovered that Nar reduced hTPC2 activity in a voltage-independent and reversible manner, with an IC50 of about 200 μM. We also succeeded in expressing the other member of the TPC family present in humans, namely hTPC1, in our plant system and verified that Nar behaved as a channel inhibitor in this case as well. Similarly, Nar reduced the activity of the endogenous Arabidopsis TPC1, thereby indicating that the mechanism is conserved from the evolutionary point of view.

Interestingly, the physical-chemical characteristics of flavonoids indicate that these compounds cross biological membranes. Therefore, Nar might permeate the cellular membrane and reach intracellular compartments such as endosomes and lysosomes in which TPC channels are localised. To assess the possible effects of Nar on TPC2 in human cells, we first tested the ability of Nar to inhibit the Ca^2+^-activated responses of HUVECs to VEGF, which are known to depend upon the NAADP/TPC2-regulated pathway^41^. Our Ca^2+^ imaging data show that Nar inhibited the increase in [Ca^2+^]_i_ elicited by both VEGF and histamine (positive control^47^) but not that elicited by ATP or Ang-1, which are known to be independent of NAADP/TPC2 (negative controls^48^).

As our previous data have shown^41^, VEGF stimulation triggers the NAADP/TPC2/Ca^2+^ cascade by inducing phosphorylation of VEGFR2 at Tyr1175. Here we show that VEGF-induced phosphorylation of VEGFR2 is not affected by Nar, thereby demonstrating that inhibition of the response pathway occurs downstream of the receptor. In another set of experiments, we further restricted the possible targets of Nar inhibition to TPC2 by stimulating the cells directly with NAADP, which is known to bind TPC2 and elicit Ca^2+^ release from acidic compartments. Ca^2+^ imaging experiments with NAADP-AM were performed in the absence of external Ca^2+^ in order to monitor the involvement from internal stores alone, thus limiting possible off-target responses. In this experimental condition, the NAADP-induced mobilization of Ca^2+^ was significantly inhibited by pretreatment with Nar. This observation strongly suggests a direct effect of Nar on TPC2 given the specificity of the agonist and the preclusion of Ca^2+^ entry through ion channels or pumps localized on the cell membrane.

The antiangiogenic effects of Nar have recently been reported to involve impairment of the synthesis of estrogen-related receptor α (ERRα) and VEGF secretion^4^, though the specific cascade of signaling events has yet to be understood. Given the involvement of NAADP/TPC2 in the response to VEGF, we explored whether inhibition of this pathway by Nar might account for the reported impairment of angiogenesis. To this end, we compared the ability of Nar to interfere with the formation of capillary-like tubes in HUVEC cultures treated either with VEGF or with Ang-1. As expected, both factors stimulated the formation of short segments, which became interconnected within hours and formed closed polygons. Interestingly, the number of closed polygons was reduced by Nar in VEGF-stimulated though not in Ang-1-stimulated samples, which allowed NAADP-insensitive Ca^2+^ stores to be ruled out as Nar targets.

In addition, we successfully tested the antiangiogenic effect of Nar in a mammalian *in vivo* model of neoangiogenesis. In a previous article by our group^41^ we showed that VEGF-treated plugs implanted in the flank of wild type mice not only failed to be vascularized if treated with the NAADP inhibitor Ned-19, but more strikingly remained avascular in TPC2 −/− mice; moreover, the specific need for functional TPC2 channels is clearly highlighted by the observation that vascularization was not inhibited in plugs from TPC1 −/− mice^41^. In our experimental murine model of matrigel plug, neo-vascularization stimulated by VEGF was observed in the control samples but failed to occur in Nar-treated plugs. We cannot exclude the possibility that targets other than TPC2 may contribute to the observed effect *in vivo* but these data open an interesting perspective towards a potential therapeutic approach.

Besides confirming the inhibitory properties of Nar in VEGF-induced angiogenesis, the data from the present study shed new light on the mechanisms underlying this event by revealing the pivotal role played by TPC2 and provide the first evidence of the antiangiogenic properties of Nar in an *in vivo* murine model.

Significant inhibitory effects were observed in our study at high Nar concentrations. In another study, which was conducted on SCID mice^52^ animal survival was unaffected by i.p. injections of doses of up to 5 mM Nar. The possible application of these data to human therapies may be hampered by problems related to the low oral bioavailability of Nar (see ref. 53 as an example to enhance the solubility and enteral uptake of Nar) and restrictions imposed by clinical trials, though these issues are beyond the scope of this study.

Nar is known to exert a wide range of effects induced by a number of mechanisms that have yet to be discovered. By contrast, a limited number of factors are known to modulate TPC2 activity, and our data shed light on a novel, naturally-occurring molecule that targets this channel which is attracting a considerable amount of interest owing to its relevance to important pathophysiological processes. The relationship we describe here between naringenin and TPC2 is therefore likely to have wider implications in systems other than the vascular system, thus representing a novel tool for experimental, and possibly even clinical, research purposes.

## Materials and Methods

### Arabidopsis protoplast transformation

*Arabidopsis thaliana tpc1–2* mutants^23^ were grown in soil in a growth chamber at 22 °C and 8 h light/16 h dark regime. Well-expanded leaves from 4 weeks old plants were used for mesophyll protoplast preparation as described in refs 44 and 54. The transient transformation was performed as described previously^35, 46^ using the pSAT-hTPC2-EGFP or pSAT-hTPC1-EGFP plasmid as reported therein. The transformed cells were maintained in the dark at 23 °C in W5 ionic solution (in mM: 154 NaCl, 125 CaCl_2_, KCl 5, MES 2, pH 5.6 with KOH) plus 50 µg/ml Ampicillin as described in ref. 44. Vacuoles were efficiently released from the protoplasts using the following vacuole release solution (in mM): 100 malic acid, 5 EGTA, 3 MgCl_2_, pH 7.5 using 160 1,3-bis(tris(hydroxymethyl)methylamino) propane (BTP), 450 mOsm with D-sorbitol.

### Patch-clamp recordings

Patch-clamp experiments on vacuoles from fluorescent protoplasts expressing the hTPC2-EGFP fusion on the tonoplast were performed in whole-vacuole configuration ≥40 h after the transformation. The bath solution (cytoplasmic side) contained (in mM): 100 NaCl, 2 MgCl_2_, 10 Hepes-Tris, pH 7.5. The pipette solution (vacuolar side) was (in mM): 100 NaCl, 2 MgCl_2_, 10 MES-Tris, pH 5.5. The bath solution and the pipette solution were adjusted to 580 mOsm and 618 mOsm by the addition of D-sorbitol. Dithiothreitol (DTT; 2 mM) was added to the vacuole release solution before the patch-clamp experiments. DTT was prepared as 1 M stock solution the day of the experiment and kept on ice until use. Nar was also prepared fresh just before use as 1000 mM stock in DMSO. Since the usual maximum concentration of Nar used in electrophysiological studies (100 μM) induced about 40% inhibition of hTPC2 and even less of AtTPC1, we decided to increase [Nar] up to 1000 μM. However, some precipitate was present in these conditions possibly slightly reducing the nominal Nar concentration. We also verified that the maximum DMSO concentration used in this study (up to 0.1%) were not effective on channel activity. PI(3,5)P_2_, purchased as dioctanyl ester (diC8) from AG Scientific or Echelon Biosciences Inc, was prepared as 1 mM stock solution and stored at −20 °C. The other chemicals (including Nar) were purchased from Sigma-Aldrich and Carl Roth.

### Analysis of electrophysiological data

Positive currents correspond to cations flowing from the cytoplasmic side to the lumen of the vacuole. Data analysis and figure preparation were done with Igor Pro software (Wavemetrics) or Photoshop (Adobe Inc.).

### Cell culture

HUVECs were obtained from Lonza Sales Ldt, cultured in EGM-2 Endothelial Cell Growth Medium-2 (Endothelial Basal Medium EBM-2 + EGM-2 Bullet Kit, Lonza), + 100 mM Penicillin/Streptomycin (Sigma), maintained at 37 °C in a humidified 5% CO_2_ incubator and were used at passage 1 to 6. Reagents used are: VEGF-A_165_ (Peprotech), Ang-1 (Peprotech), Histamine (Sigma), ATP (Sigma), Ned-19 (Tocris Bioscience); NAADP-AM was a generous gift of G. C. Churchill (Oxford University).

### Calcium imaging

Cells were incubated in EGM-2 containing 3.5 µM Fura-2-AM (Invitrogen) for 1 h at 37 °C and then rinsed with either Hanks’ Balanced Salt Solution (HBSS, Sigma) or Ca^2+^ free Krebs Henseleit Hepes buffer (KHH). Dishes were placed into a culture chamber kept at 37 °C controlled temperature on the stage of an inverted microfluorimeter (Nikon TE2000E) connected to a cooled CCD camera (512B Cascade, Princeton Instruments). Samples were illuminated alternately at 340 and 380 nm using a random access monochromator (Photon Technology International) and emission was detected using a 510 nm emission filter. Images were acquired (1 ratio image/s) using Metafluor software (Universal Imaging Corporation). Calibration of the signal was obtained at the end of each experiment by maximally increasing intracellular Ca^2+^-dependent Fura-2 fluorescence ratio (340/380) with 5 µM ionomycin (ionomycin calcium salt from Streptomyces conglobatus, Sigma) followed by recording minimal ratio in Ca^2+^-free medium. [Ca^2+^]_i_ was calculated according to previously described formulas^55^.

### Western Blot

HUVECs were first starved in EBM-2 + 2% FBS for 4 h and then incubated with Nar for 30 min or with TSU-68 (SU6668, Selleckchem), an inhibitor of VEGFR2 tyrosine kinase activity, for 1 h before VEGF stimulation for 15 min. The primary antibody used was anti-Phospho-VEGF Receptor 2 (Tyr1175) (Cell Signaling Technology) revealed by Goat Anti-Rabbit IgG (H+L)-HRP Conjugate (BIO-RAD). To ensure equal loading, membranes were reprobed with Monoclonal Anti-α-Tubulin antibody (Sigma) followed by Goat Anti-Mouse IgG (H+L)-HRP Conjugate (BIO-RAD). The intensity of western blot bands was quantified by Image Lab 5.2.1 software (BIO-RAD) from three independent experiments.

### *In vitro* angiogenesis assay

Capillary-like endothelial tube formation was evaluated by an *in vitro* angiogenesis assay. 130 µl Matrigel Basement membrane Matrix Growth factor Reduced (BD Biosciences) was added to each well of pre-cooled 24-well tissue culture plates. Pipette tips and matrigel solution were kept cold during the procedure to avoid solidification. The plates were incubated for 1 h at 37 °C to allow matrix solution to solidify. 4 × 10^4^ HUVECs in a final volume of 500 µl EBM-2 + 2% FBS were seeded in each matrix-coated well. Cells were pretreated with 500 µM or 1000 µM Nar or with vehicle alone and stimulated with 100 ng/ml VEGF for 3–4 hours at 37 °C. Cells stimulated with 100 ng/ml Ang-1 for 2–3 hours, pretreated with 500 µM Nar or 100 µM Ned-19, were seeded in EGM-2. Tube formation was inspected under an inverted microscope (Nikon Eclipse TS100) and images were acquired by a digital camera (Nikon Ds Fi2, Nis elements F 4.00.00 software). The closed polygons formed in at least six random view microscopic fields per well were counted and values averaged.

### *In vivo* angiogenesis assay

The ability of Nar to modulate VEGF-induced neovascularization was tested by matrigel plug assay. Matrigel (600 μl, BD Biosciences) supplemented with heparin (32 U/ml, Schwarz Pharma S.p.A), VEGF (100 ng/ml, Peprotech) or Angiopoietin1 (150 ng/ml, Peprotech), TNF-α (2 ng/ml, R&D Systems) and ±1000 μM Nar was injected subcutaneously into C57BL/6 mice flanks where it rapidly formed a gel. The negative controls contained heparin alone, the positive controls heparin plus VEGF and TNF-α. In this assay, cells from the surrounding tissues migrate into the matrigel plug and form vascular structures connected to the mouse blood vessels. After 5 days, mice were sacrificed by CO_2_ asphyxia and the angiogenic response was evaluated by macroscopic analysis at necropsy and by measurement of the hemoglobin content in the matrigel plug. Hemoglobin was mechanically extracted in water and measured using the Drabkin method by spectrophotometrical analysis (Sigma) at 540 nm. The values were expressed as optical density/100 mg matrigel.

The animals were housed in the accredited animal facility of the Department of Anatomy, Histology, Forensic Medicine and Orthopaedics, Unit of Histology and Medical Embryology, Sapienza University of Rome, in individual cages, in an environmentally controlled room (23 °C, 12 h light-dark cycle) and provided with food and water *ad libitum*. All of the procedures were approved by the Italian Ministry for Health and conducted according to Italian law.

### Statistical analysis

Data are presented as the mean ± s.e.m. of results from at least three independent experiments. Student’s t test was used for statistical comparison between means where applicable. *P < 0.05; **P < 0.01; ***P < 0.001.

### Ethical Standards

All the experiments described in this manuscript fully comply with the current laws of Italy.

## Electronic supplementary material

Supplemental Figures 1, 2, 3, 4, 5 and 6
