# Supplementary material for: Naringenin Impairs Two-Pore Channel 2 Activity And Inhibits VEGF-Induced Angiogenesis
Source: Sci Rep. 2017 Jul 11;7:5121. doi: 10.1038/s41598-017-04974-1 (PMC5505983; doi:10.1038/s41598-017-04974-1)
Supplement: Supplementary file 1 — Supplemental Figures 1, 2, 3, 4, 5 and 6 [file 41598_2017_4974_MOESM1_ESM.pdf]

NARINGENIN IMPAIRS TWO PORE CHANNEL-2 ACTIVITY AND INHIBITS VEGF-INDUCED ANGIOGENESIS

Irene Pafumi, Margherita Festa, Francesca Papacci, Laura Lagostena, Cristina Giunta, Vijay Gutla, Laura Cornara, Annarita Favia, Fioretta Palombi, Franco Gambale, Antonio Filippini and Armando Carpaneto

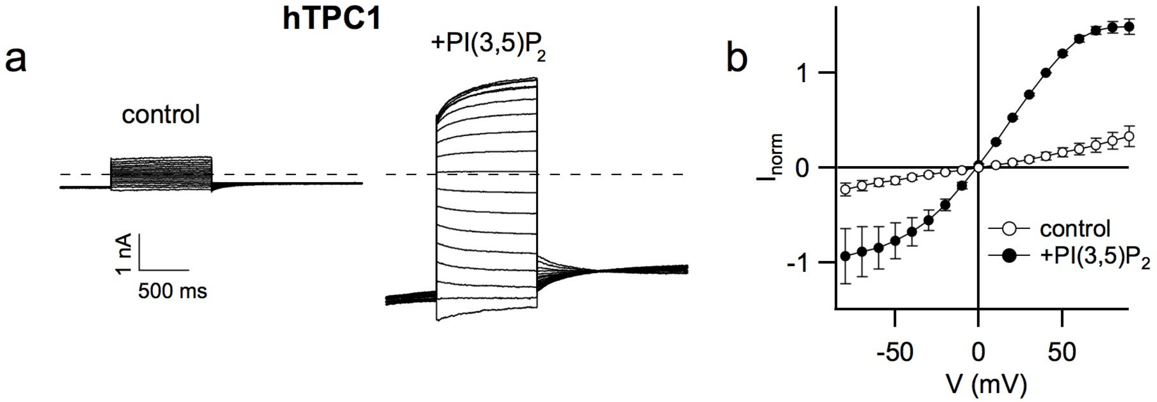

Suppl. Fig. 1: the human TPC1 channel is expressed in TPC-free Arabidopsis vacuoles
(a) Currents recorded in the absence (left) and in the presence (right) of 90 nM PI(3,5)P_2 added in the cytosolic bath solution. Currents were elicited by voltages ranging to -90 to +80 mV in 10 mV step. Holding and tail voltages were respectively of -70 and -50 mV. (b) Steady-state currents obtained as the mean values of the last 100 ms recording were plotted against the applied voltages. Empty and filled symbols indicated the absence and the presence of 90 nM PI(3,5)P2. Data, shown as mean± s.e.m., were from 7 different vacuoles.

Suppl. Fig. 1

NARINGENIN IMPAIRS TWO PORE CHANNEL-2 ACTIVITY AND INHIBITS VEGF-INDUCED ANGIOGENESIS

Irene Pafumi, Margherita Festa, Francesca Papacci, Laura Lagostena, Cristina Giunta, Vijay Gutla, Laura Cornara, Annarita Favia, Fioretta Palombi, Franco Gambale, Antonio Filippini and Armando Carpaneto

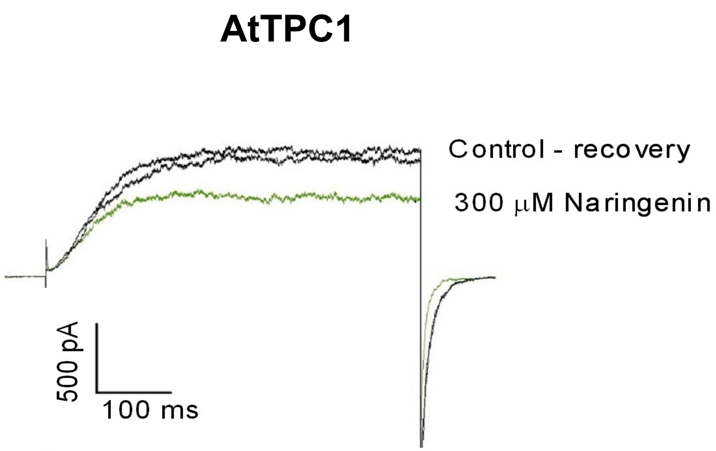

Suppl. Fig. 2: Naringenin inhibits the *Arabidopsis thaliana* TPC1
AtTPC1 channel response to voltage stimulation at V= +80 mV in control, in the presence of 300 µM cytosolic Nar and in recovery conditions in vacuoles from mesophyll cells of *Arabidopsis thaliana* plants. Ionic solutions: 200 mM KCl, 2 mM $MgCl_2$, 2 mM $CaCl_2$, 10 mM MES/Tris, pH 5.5 in the pipette; 100 mM KCl, 2 mM $MgCl_2$, 1 mM $CaCl_2$, 1 mM dithiothreitol (DTT), and 10 mM HEPES/Tris, pH 7.5 in the bath; osmolarity adjusted to 600 mOsm by the addition of D-sorbitol. Similar experiments were performed from at least 15 different vacuoles.

Suppl. Fig. 2

NARINGENIN IMPAIRS TWO PORE CHANNEL-2 ACTIVITY AND INHIBITS VEGF-INDUCED ANGIOGENESIS

Irene Pafumi, Margherita Festa, Francesca Papacci, Laura Lagostena, Cristina Giunta, Vijay Gutla, Laura Cornara, Annarita Favia, Fioretta Palombi, Franco Gambale, Antonio Filippini and Armando Carpaneto

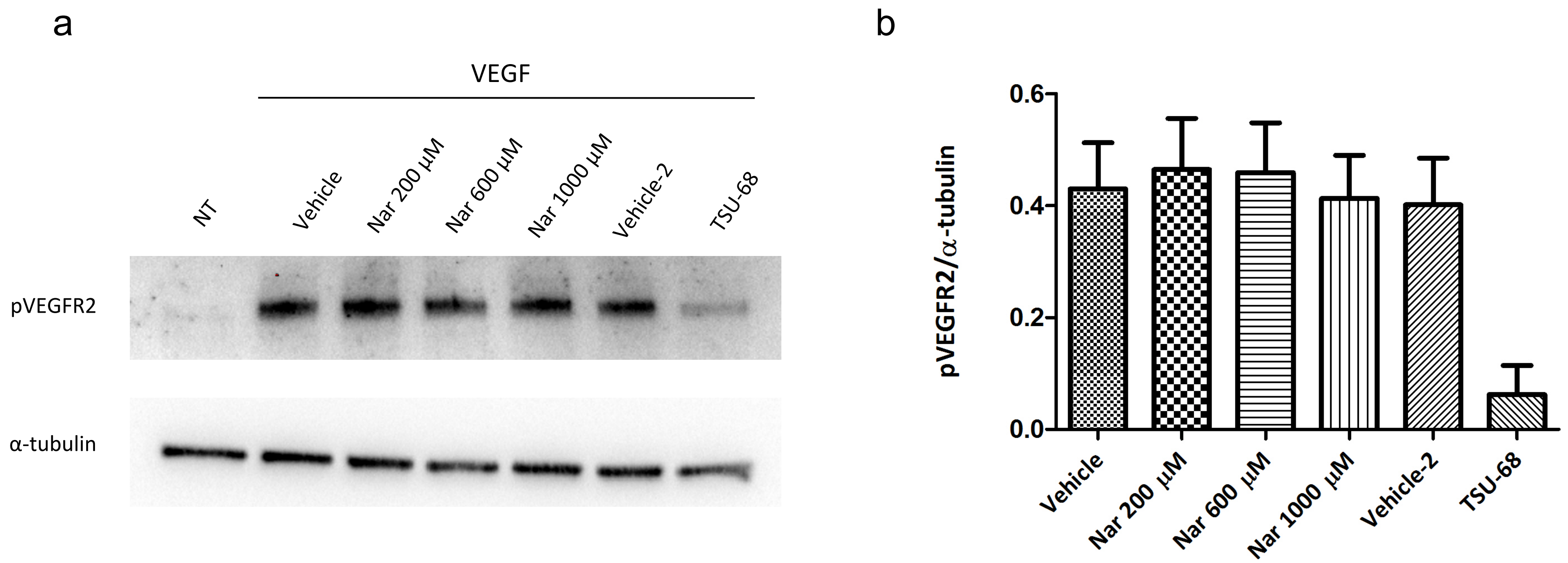

Suppl. Fig. 3: Phosphorylation of VEGFR2 is not affected by naringenin treatment.
Phosphorylation state of VEGFR2 at Tyr1175, evaluated by western blot in untreated (NT) or VEGF-treated HUVECs. Cells were preincubated with Nar (200 µM, 600 µM and 1000 µM) or vehicle for 30 min, or with TSU-68 (2,1 µM), or its control (vehicle-2) for 1h. Samples were then stimulated with 100 ng/ml VEGF for 15 min (a). The intensity of pVEGFR2 bands was quantified and normalized to α-tubulin content. Data in bar chart represent mean ± s.e.m. from three independent experiments (b). As apparent, VEGF-induced receptor phosphorylation is significantly inhibited by TSU-68 but not by Nar.

Suppl. Fig. 3

NARINGENIN IMPAIRS TWO PORE CHANNEL-2 ACTIVITY AND INHIBITS VEGF-INDUCED ANGIOGENESIS

Irene Pafumi, Margherita Festa, Francesca Papacci, Laura Lagostena, Cristina Giunta, Vijay Gutla, Laura Cornara, Annarita Favia, Fioretta Palombi, Franco Gambale, Antonio Filippini and Armando Carpaneto

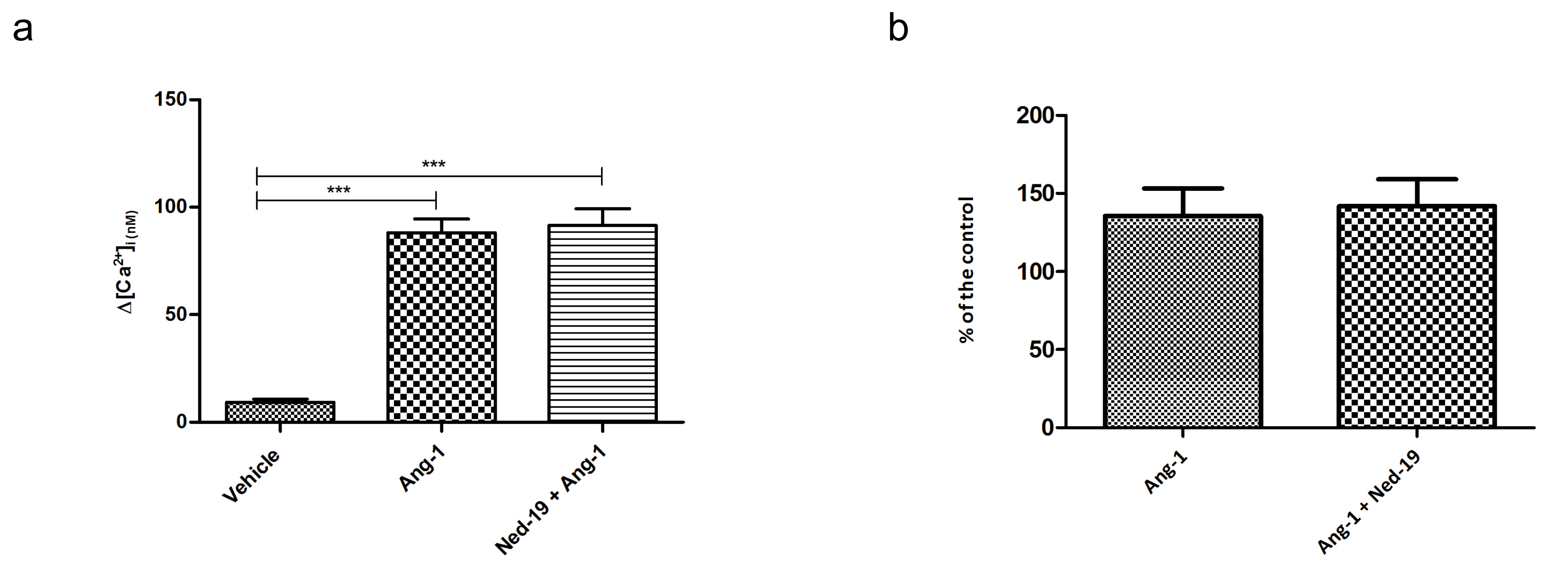

Suppl. Fig. 4: Neither $[Ca^{+2}]_i$ increase nor the formation of capillary-like tubes stimulated by Ang-1 are affected by Ned-19.
(a,b) Cells were pretreated for 30 min with 100 µM Ned-19 and stimulated with 100 ng/ml Ang-1. (a) Bar chart showing maximum $[Ca^{+2}]_i$, ***P<0.001; (b) Cells were plated in Matrigel-coated dishes and incubated for 2-3 h in EGM-2 supplemented with Ang-1 or Ang-1 + Ned-19. Quantitative evaluation of tube formation as the number of closed polygons formed in 9 fields for each experimental condition. Data in bar charts represent percentage of the control (mean ± s.e.m. of three independent experiments).

Suppl. Fig. 4

NARINGENIN IMPAIRS TWO PORE CHANNEL-2 ACTIVITY AND INHIBITS VEGF-INDUCED ANGIOGENESIS

Irene Pafumi, Margherita Festa, Francesca Papacci, Laura Lagostena, Cristina Giunta, Vijay Gutla, Laura Cornara, Annarita Favia, Fioretta Palombi, Franco Gambale, Antonio Filippini and Armando Carpaneto

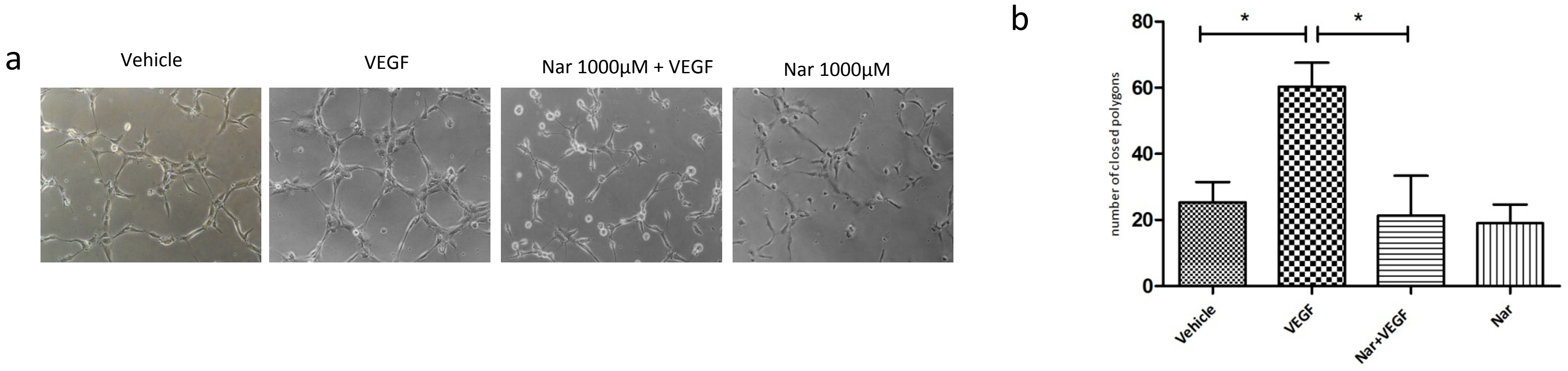

Suppl. Fig. 5: Naringenin impairs VEGF- induced capillary tube formation *in vitro.*
(a) Representative images of one of three independent experiments. HUVECs were plated in Matrigel-coated dishes and incubated for 4 h in EBM-2+2%FBS supplemented or not with VEGF or Nar, or in medium containing both VEGF and Nar. Each condition was tested in triplicate for each individual experiment. (b) Quantitative evaluation of tube formation as the number of closed polygons formed in 6 fields for each experimental condition. Data in bar chart represent mean ± s.e.m. of three independent experiment. *P<0.05.

NARINGENIN IMPAIRS TWO PORE CHANNEL-2 ACTIVITY AND INHIBITS VEGF-INDUCED ANGIOGENESIS

Irene Pafumi, Margherita Festa, Francesca Papacci, Laura Lagostena, Cristina Giunta, Vijay Gutla, Laura Cornara, Annarita Favia, Fioretta Palombi, Franco Gambale, Antonio Filippini and Armando Carpaneto

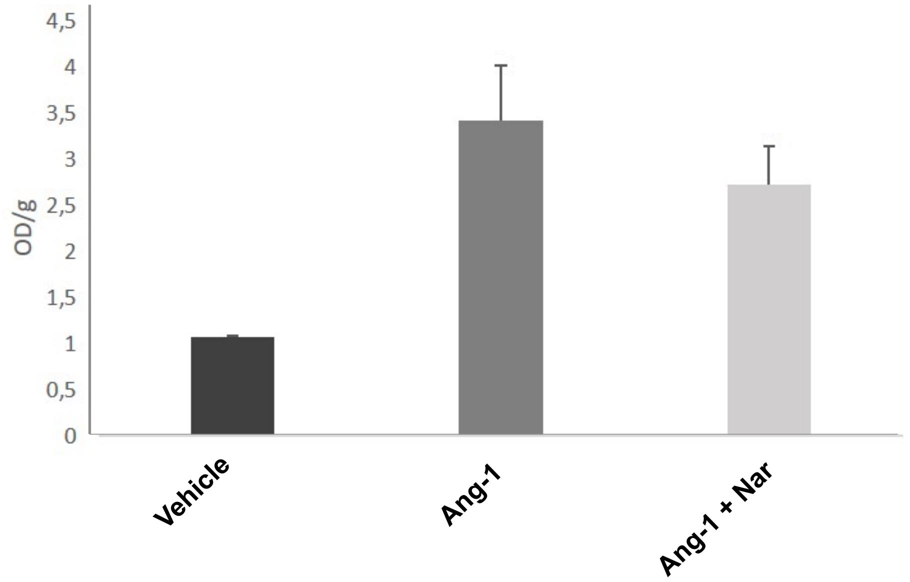

Suppl. Fig. 6: Naringenin does not impair *in vivo* vascularization induced by Ang-1

In vivo vessel formation was assessed after subcutaneous injection of 5 weeks old male/female C57BL/6 mice with Matrigel plugs containing either vehicle or Ang-1 (150 ng/ml) or Ang-1 plus 1000 µM Nar. Five days after injection the mice were sacrificed and plug vascularization was evaluated as hemoglobin content expressed as absorbance (OD)/1 g matrigel plug. Hemoglobin content in Ang-1 plugs (n=8) did not significantly differ from that in Ang-1 + Nar plugs (n=8) (P>0.2).

Suppl. Fig. 6
